# 

**Published:** 1923-10

**Authors:** 


CONTENTS.

*Page*

A Film Method for Determining the Reaction of the Liquids of the Body by Indicators. By GEORGE A. BUCKMASTER, M.A., M.D. Oxon. ... 175

On the Diagnosis and Treatment of Diabetes Mellitus, with Special Reference to the Usages of Insulin. By A. T. TODD, M.B., Ch.B. Edin., M.R.C.P. Lond. ... 182

Disorders of Deglutition. By J. P. I. HARTY, B.A., M.B., B.Ch. F.R.C.S. ... 195

Reviews of Books ... 204

Editorial Notes ... 212

Obituary: EDWARD RATCLIFFE HOLBOROW, M.B., B.S. Lond., F.R.C.S. Ed. ... 214

